# Bacterial Diversity of Intestinal Microbiota in Patients with Substance Use Disorders Revealed by 16S rRNA Gene Deep Sequencing

**DOI:** 10.1038/s41598-017-03706-9

**Published:** 2017-06-15

**Authors:** Yu Xu, Zhenrong Xie, Huawei Wang, Zongwen Shen, Youbing Guo, Yunhong Gao, Xin Chen, Qiang Wu, Xuejun Li, Kunhua Wang

**Affiliations:** 1 Yunnan Institute of Digestive Disease, the First Affiliated Hospital of Kunming Medical University, Kunming, 650032 Yunnan China; 2 Department of Gastrointestinal Surgery, the First Affiliated Hospital of Kunming Medical University, Kunming, 650032 Yunnan China; 3 Kunming Engineering Technology Center of Diagnosis and Treatment of Digestive Diseases, Kunming, 650032 Yunnan China; 4 Department of Reproduction and Genetics, the First Affiliated Hospital of Kunming Medical University, Kunming, 650032 Yunnan China; 5 Yunnan Drug Enforcement Commission Office, Kunming, 650032 Yunnan China; 6 Yunnan Drug Enforcement Administration, Kunming, 650032 Yunnan China

## Abstract

Substance abuse and addiction are worldwide concerns. In China, populated with over 1.3 billion people, emerging studies show a steady increase in substance abuse and substance-related problems. Some of the major challenges include a lack of an effective evaluation platform to determine the health status of substance-addicted subjects. It is known that the intestinal microbiota is associated to the occurrence and development of human diseases. However, the changes of bacterial diversity of intestinal microbiota in substance-addicted subjects have not been clearly characterized. Herein, we examined the composition and diversity of intestinal microbiota in 45 patients with substance use disorders (SUDs) and in 48 healthy controls (HCs). The results show that the observed species diversity index and the abundance of *Thauera*, *Paracoccus*, and *Prevotella* are significantly higher in SUDs compared to HCs. The functional diversity of the putative metagenomes analysis reveals that pathways including translation, DNA replication and repair, and cell growth and death are over-represented while cellular processes and signaling, and metabolism are under-represented in SUDs. Overall, the analyses show that there seem to be changes in the microbiota that are associated with substance use across an array of SUDs, providing fundamental knowledge for future research in substance-addiction assessment tests.

## Introduction

Substance abuse and addition cause issues to both personal and family health, which causes a serious problem in China^1^. Substance addiction has been shown to lead to increased violence, prostitution, substance-related sexually transmitted diseases (i.e. acquired immune deficiency syndrome: AIDS) and death. Yunnan is a Chinese border province, geographically adjacent to Southeast Asia’s “Golden Triangle”, known as the world hub for opium and heroin manufacturing and trafficking. Over the past two decades, Yunan province has experienced a remarkable rise in illegal substance use. At the end of 2014, it was estimated that there were 2.9 million substance-addicts in China and over 188,000 registered substance addicts in Yunnan province^2^.

Substance addiction is a chronic disease, in which addicted patients compulsively seek and take substances, even though they are aware of its harmful consequences^3^. The substance-addicted patients often have co-occurring medical conditions, including malnutrition, AIDS, Hepatitis C, cardiovascular diseases, stroke, and mental disorders. In addition, most of substance abusers are often addicted to alcohol and tobacco. To treat substance addiction and to help former substance addicts return back to society, community-based drug rehabilitation centers have been developed in Yunnan province. However, a major challenge is a lack of effective evaluations/tests to determine the health status of the patients and the effects of drug rehabilitation, both physically and mentally.

The microorganisms that live within and on the surface of the human body are composed of over 100 trillion species^4^. It is estimated to outnumber human body cells by at least an order of magnitude. Recent findings suggest that these microorganisms play significant roles in maintaining human health, and the microbiota: the compositions of the microorganisms that directly reflect health status of an individual^5, 6^. For example, emerging evidences show that the intestinal microbiota contributes to fat deposition and glucose metabolism, because certain changes in the intestinal microbiota were observed in obese patients^7, 8^. Therefore, intestinal microbiota diversity has emerged as an indicator of overall health of the host. In addition, low community richness has been correlated with metabolic disorders (i.e. adiposity, insulin resistance, and overall inflammatory phenotypes) and gastrointestinal conditions (i.e. inflammatory bowel disease, colorectal cancer, and irritable bowel syndrome)^9^. Various external variables such as stress, probiotic or antibiotic use, and alcohol consumption have been found to instigate changes in the human microbiota^10, 11^.

In this study, we examined the composition and the dynamics of the intestinal microbiota of 45 patients with substance use disorders (SUDs) and 48 healthy controls ﻿(HCs) in order to determine whether there is an association between substance abuses and/or the length of substance addiction with the changes of intestinal microbiota. To our knowledge, this is the first study comprehensively comparing the composition and diversity of intestinal microbiota between SUDs and HCs.

## Results

### Characteristics of Participants in the Study

In total 101 male subjects who take substances including heroin, ice, ephedrine, heroin + ephedrine, and heroin + ice provided fecal samples in this study. 8 subjects were excluded due to the intake of antibiotics before sampling. It is noticeable that all subjects with substance use disorders are involved in daily smoking and drinking. The detailed demographic and clinical characteristics were summarized in Table 1.

**Table 1:** Characteristics of 101 Participants.

	HCs	SUDs
**Gender**	Male (n = 51)	Male (n = 50)
**Age at collection**		
** Mean ± SD**	25 ± 3	32 ± 7
** Range**	20~37	18~46
**Substance_Time** (**month**)		
** Mean ± SD**	NA	54 ± 69
** Range**		3~348
**Substance_Type**		
** Heroin**	NA	26
** Methamphetamine**		15
** Ephedrine**		4
** Others**		5
**Substance_Manner**		
** Snorting**	NA	45
** Injection**		5
**Re-addiction Times**		
** 1st time**	NA	17
** 2nd time**		21
** More than twice**		12

Data are shown as median (interquartile range).

### Substance addition affects intestinal microbiota in SUDs

First, we performed a principle component analysis (PCA) to distinguish the differences among subjects addicted to different groups of substances, including 45 SUDs and 48 HCs (Supplementary Figure S1). We found that there is no significant clustering based on substance types, with HC cluster far away from samples of SUDs. Therefore, we divided the subjects into two groups, “healthy” vs. “addicted”- SUDs with alcohol and tobacco as additional substances.

Then we analyzed the intestinal microbiota diversity on all 45 SUDs and 48 HCs, and tested whether intestinal microbiota diversity could be related to substance addiction. The overall intestinal microbiota profile was obtained using deep sequencing of the 16S rRNA gene, and microbial diversity was then analyzed.

**Figure 1:**
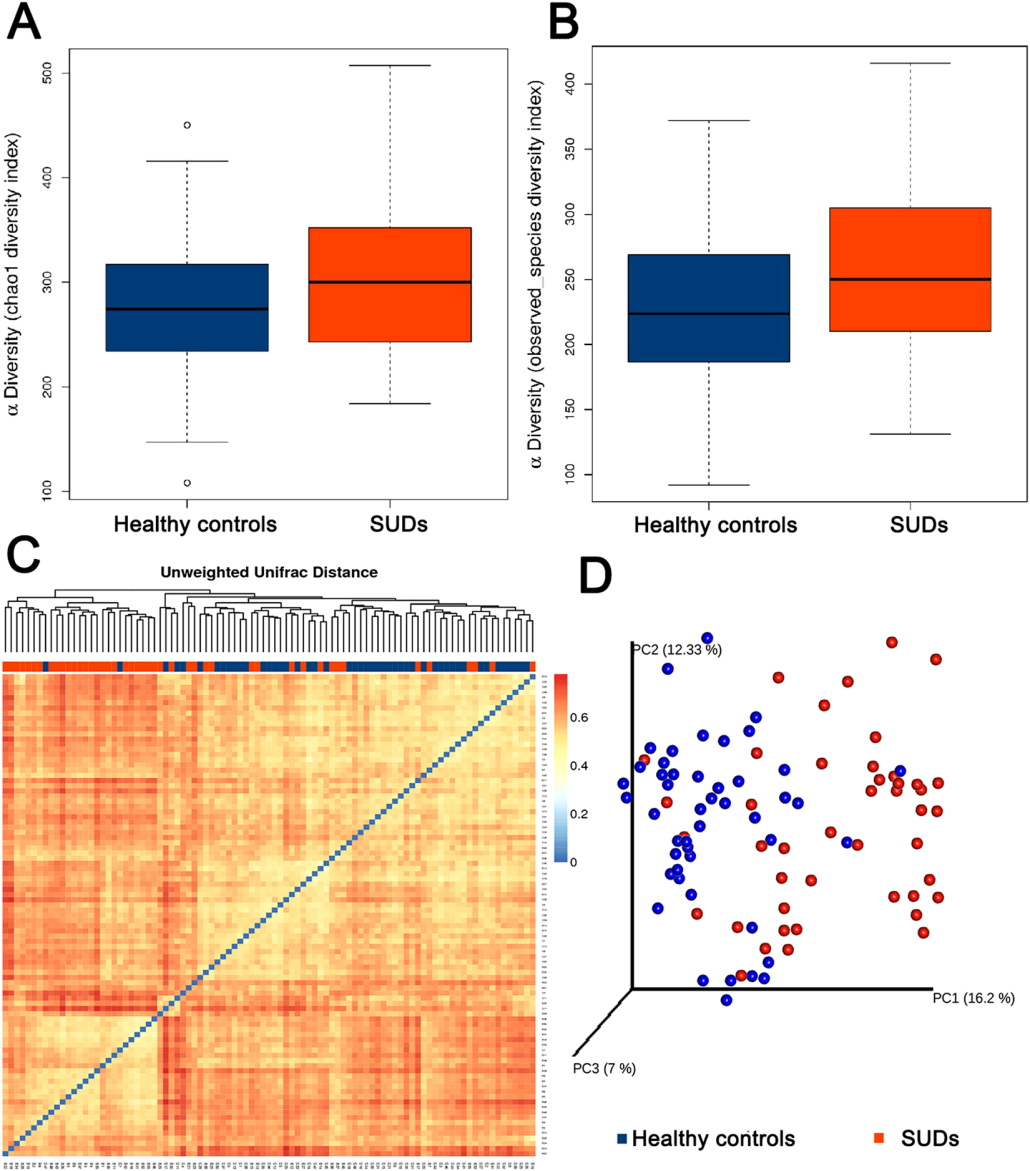
Analysis of alpha diversity in HC (blue) vs SUDs (orange) predicted diversity by Chao 1 estimator (**A**), p = 0.11 and observed species (**B**), p = 0.03. Beta diversity measures in SUDs vs HCs. (**C**) Hot map was performed basing on the unweighted-unifrac distances matrix using the R package “vegan”, orange for the SUDs and blue for the HCs. (**D**) Principal Coordinate Analysis (PCoA) and UPGMA tree of unweighted-unifrac distances of samples, blue for the HCs and red for the SUDs (*adonis* test, R2 = 0.067, Pr (>F) = 0.001).

The alpha diversity indices of Chao1 and observed species diversity are shown in Fig. 1. The Chao1 diversity index was higher in SUDs compared to the HCs, but there were no significant differences between the groups by *t-test* (Fig. 1A). However, the observed species diversity index was significantly different between all 48 SUDs and all 45 HCs (Fig. 1B, *p* = 0.03). To exclude potential effects of age on the profiles of microbial diversity in SUDs, additional analysis was applied to 29 SUDs and 28 age-matched HCs aging from 19 to 37 (Table 2). Both Chao1 diversity index and observed species diversity index were higher in SUDs compared to HCs, but there were no significant differences between groups through a *t-test* (Data not shown). Beta diversity was further evaluated with unweighted-UniFrac analysis, which showed a global difference in microbial community composition from 48 SUDs from 45 HCs (Fig. 1C). Furthermore, UniFrac-based Principal Coordinate Analysis (PCoA) showed that samples were clustered by subject (Fig. 1D, R2 = 0.067, Pr (>F) = 0.001). We also performed a weighted-UniFrac PCoA analysis with R2 = 0.017, Pr (>F) = 0.187 (Supplementary Figure S2). These results suggest that factors associated with substance addiction may strongly influence the diversity of intestinal microbiota.

**Table 2:** Characteristics of 57 Participants.

	HCs	SUDs
**Gender**	Male (n = 28)	Male (n = 29)
**Age at collection**		
** Mean ± SD**	27 ± 3	29 ± 5
** Range**	25~37	19~36
**Substance_Time** (**month**)		
** Mean ± SD**	NA	37 ± 42
** Range**		8~192
**Substance_Type**		
** Heroin**	NA	15
** Methamphetamine**		10
** Ephedrine**		2
** Others**		2
**Substance_Manner**		
** Snorting**	NA	26
** Injection**		3
**Re-addiction Times**		
** 1st time**	NA	8
** 2nd time**		15
** More than twice**		6

Data are shown as median (interquartile range).

### Bacterial Abundance and Distribution in SUDs vs. HCs

**Figure 2:**
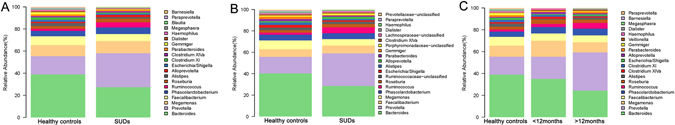
Genus-level taxonomic distribution of intestinal microbiota and top 20 genera in (**A**) 45 SUDs vs 48 HCs, (**B**) 29 age-matched SUDs vs 28 age-matched HCs, and (**C**) long-term SUDs vs short-term SUDs. Stacked columns for each of the group show the mean of abundance of a given genus as a percentage of the total bacterial sequences in the corresponding group.

We then analyzed the intestinal microbiota abundance and distribution on the 45 SUDs and the 48 HCs, and tested whether intestinal microbiota abundance and distribution could be related to substance addiction. Nonmetric multidimensional scaling revealed that the bacterial profiles of SUDs differed from those of HCs (Fig. 2). We investigated the potential bacterial groups responsible for the changes observed in the profiles of SUDs and used Kruskal-Wallis ANOVA test to see if there is any particular OTU of interest.

**Figure 3:**
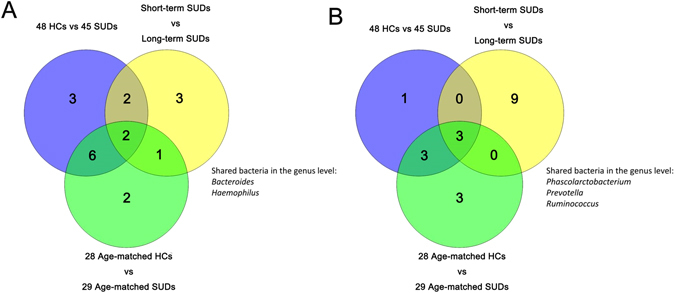
Three-group shared bacteria in the VENN. Bacteria from *Bacteroides*, and *Haemophilus* (**A**) were consistently less abundant whereas the number of bacteria from *Prevotella*, *Phascolarctobacterium* and *Ruminococcus* (**B**) were consistently increased in SUDs vs HCs, and in long-term SUDs vs short-term SUDs.

At the genus level, bacteria from *Bacteroides* (24.20% vs 33.97%), *Faecalibacterium* (5.62% vs 6.93%), *Alistipes* (1.21% vs 2.49%), *Gemmiger* (0.49% vs 1.68%), *Clostridium XI* (0.77% vs 1.83%), *Escherichia*/*Shigella* (0.99% vs 1.65%), *Dialister* (0.43% vs 0.98%), *Paraprevotella* (0.19% vs 0.72%), *Megasphaera* (0.23% vs 0.70%), *Haemophilus* (0.37% vs 0.80%), *Parabacteroides* (1% vs 1.29%), *Barnesiella* (0.26% vs 0.53%), and *Blautia* (0.44% vs 0.48%) were less abundant whereas those from *Prevotella* (27.20% vs 14.42%), *Ruminococcus* (3.97% vs 1.64%), *Phascolarctobacterium* (5.37% vs 4.15%), *Alloprevotella* (1.76% vs 0.91%), *Megamonas* (9.64% vs 8.86%), *Roseburia* (2.94% vs 2.16%), and *Clostridium XlVa* (1.19% vs 1.10%) were more abundant in SUDs compared to HCs (Fig. 2A and Table S1). The same analysis was also applied to the selected 29 SUDs and 28 age-matched HCs (Fig. 2B and Table S2): at the genus level, bacteria from *Bacteroides* (26.62% vs 37.02%), *Megamonas* (5.27% vs 7.35%), *Gemmiger* (0.44% vs 2.06%), *Escherichia*/*Shigella* (1.10% vs 2.45%), *Alistipes* (1.17% vs 2.22%), *Parabacteroides* (1.07% vs 1.80%), *Paraprevotella* (0.23% vs 0.76%), *Dialister* (0.47% vs 1.00%), *Haemophilus* (0.50% vs 0.90%), *Veillonella* (0.57% vs 0.69%), *Blautia* (0.36% vs 0.55%), *Megasphaera* (0.25% vs 0.51%), *Parasutterella* (0.19% vs 0.47%), and *Clostridium XlVa* (1.05% vs 1.07%) were less abundant whereas those from *Prevotella* (27.80% vs 13.40%), *Ruminococcus* (5.05% vs 1.80%), *Roseburia* (2.87% vs 2.04%), *Alloprevotella* (1.73% vs 0.99%), *Faecalibacterium* (6.60% vs 6.41%), and *Phascolarctobacterium* (4.95% vs 4.79%) were more abundant in SUDs compared to HCs. The majority of the grouped bacteria were observed in both comparisons, *Clostridium XI*, *Megasphaera*, *Barnesiella*, and *Blautia*was only shown in the comparison between SUDs and HCs, suggesting that age may be an affecting factor for the abundance and distribution of these four bacteria. To further determine whether bacterial profile changes during long-term addiction, we compared intestinal microbiota composition of those SUDs with a longer history of substance use to those of a shorter one. At genus level, the abundance of *Prevotella* (14.42% vs 18.26% vs 30.83%), *Ruminococcus* (1.64% vs 2.45% vs 4.59%), *Alistipes* (2.49% vs 0.54% vs 1.49%), *Parabacteroides* (1.29% vs 0.41% vs 1.23%), *Escherichia*/*Shigella* (1.65% vs 0.73% vs 1.10%), *Megasphaera* (0.70% vs 0.02 vs 0.31%), *Dialister* (0.98% vs 0.41% vs 0.44%), *Barnesiella* (0.53% vs 0.14% vs 0.31%), *Phascolarctobacterium* (4.15% vs 5.36% vs 5.37%), *Gemmiger* (1.68% vs 0.42% vs 0.52%), and *Paraprevotella* (0.72% vs 0.14% vs 0.21%) were increased, whereas *Bacteroides* (33.97% vs 31.18% vs 21.36%), *Megamonas* (8.86% vs 13.14% vs 8.22%), *Roseburia* (2.16% vs 3.67% vs 2.64%), *Haemophilus* (0.80% vs 0.99% vs 0.12%), *Clostridium XI* (1.83% vs 1.38% vs 0.53%), *Clostridium XlVa* (1.10% vs 1.76% vs 0.96%), *Blautia* (0.48% vs 0.71% vs 0.33%) and *Faecalibacterium* (6.93% vs 5.86% vs 5.53%) were decreased in long-term SUDs compared to the short-term SUDs (Fig. 2C and Table S3). Among all three-comparison groups, bacteria from *Bacteroides* and *Haemophilus* were consistently less abundant whereas the number of bacteria from *Prevotella*, *Phascolarctobacterium* and *Ruminococcus* were consistently increased in SUDs vs HCs, and in long-term SUDs vs short-term SUDs (Fig. 3 and Table S1–3). These results suggest that the abundance of *Bacteroides* and *Prevotella* inintestinal microbiota that have significant difference compared to controlscould be utilized as potential biomarkers for SUDs and predict the status or length of substance use. In addition, bacteria from *Alloprevotella*, *Clostridium XlVa* and *Roseburia* were more abundant in overall SUDs comparing to those in HCs from the very beginning. However, the abundance of these 3 bacteria decreased in SUDs over a 12-month period. In contrast, bacteria from *Alistipes* and *Paraprevotella*, which were identified with lower abundance in overall SUDs comparing to controls, were increased in long-term SUDs. These results suggest that the abundance of bacteria from *Alloprevotella*, *Clostridium XlVa*, *Roseburia*, *Alistipes* and *Paraprevotella* changed in those SUDs and could be potentially recovered after longer period of substance use and addiction.

### Differential Microbiota Compositions and Functionality

**Figure 4:**
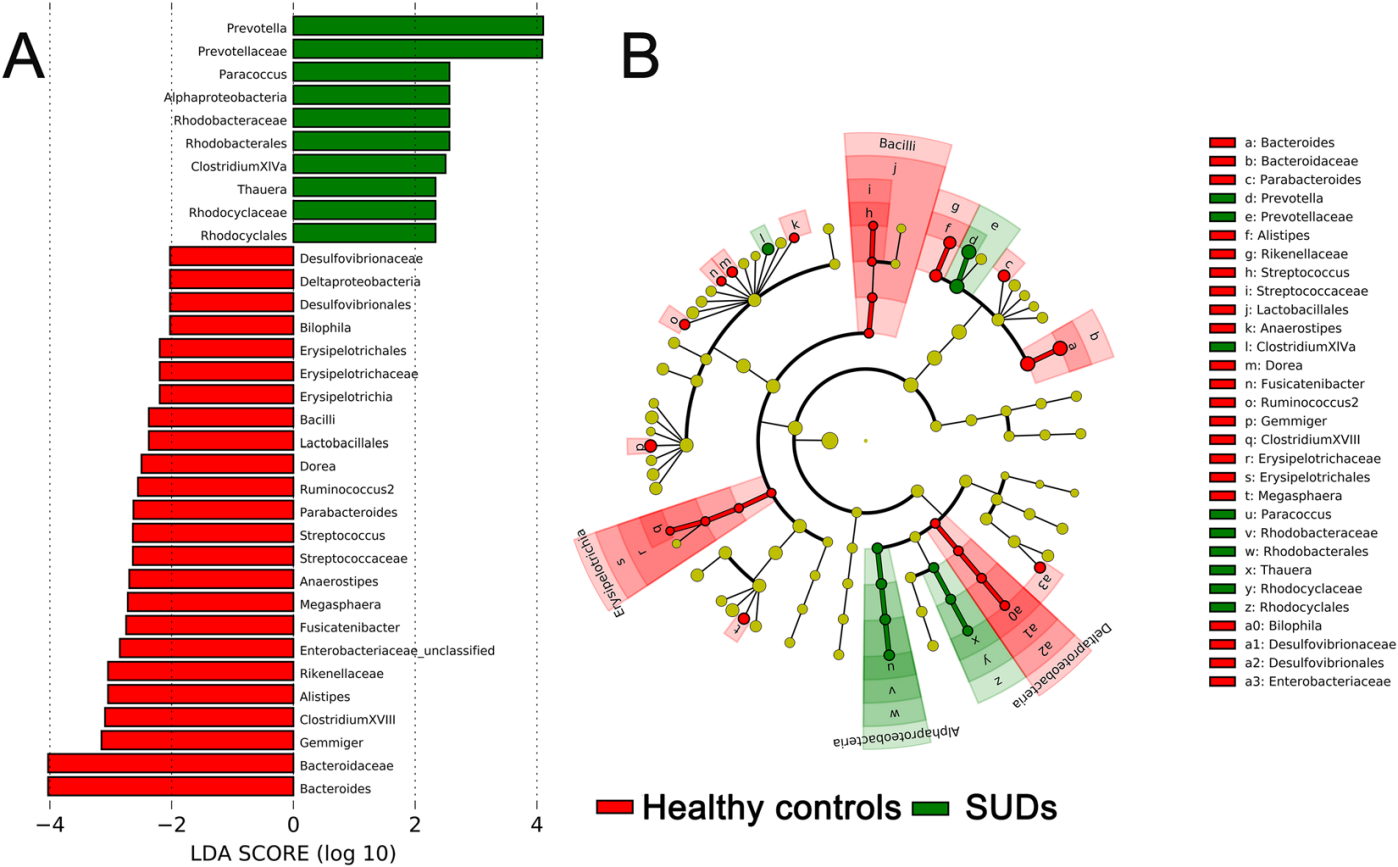
Taxonomic biomarkers. (**A**) Linear discriminative analysis (LDA) effect size LEfSe analysis between the HCs (red) and SUDs (green). (**B**) Cardiogram showing differentially abundant taxonomic clades with an LDA score > 2.0 among cases and controls, p < 0.05.

At the same time, we found significant differences in the community compositions between all 45-tested SUDs and 48 HCs. As shown in Fig. 4, there were eightsignificantly different families, which were composed by *Prevotellaceae*, *Rhodobacteraceae* and *Rhodocyclaceae* that are enriched in the SUDs group, and *Bacteroidaceae*, *Rikenellaceae*, *Streptococcaceae*, *Erysipelotrichaceae*, and *Desulfovibrionaceae* that are enriched in the HC group. The microbial composition was also significantly different at the genus level, with eighteen significantly different genera between groups. These differentially abundant taxa can be considered as potential biomarkers (LDA score > 2.0, *p* < 0.05) (Fig. 4A). *Bacilli* (p = 0.0316), *Alphaproteobacteria* (p < 0.0001), *Deltaproteobacteria* (p = 0.0020) and *Erysipelotrichia* (p = 0.0133) representation of significantly different at the class level between two groups was performed by LEfSe (Linear discriminant analysis Effect Size) (Fig. 4B)^12^.

**Figure 5:**
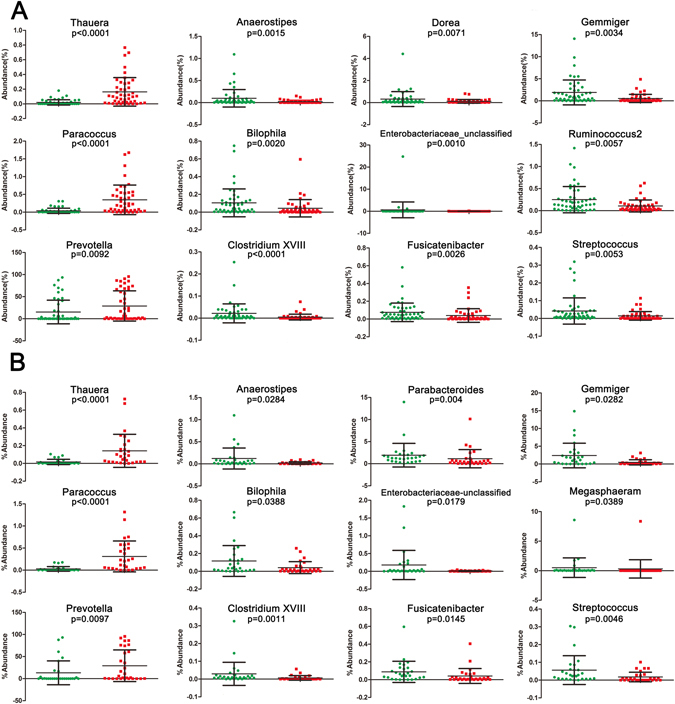
Scatterplots of bacterial taxa indicative of SUDs. HC samples are shown as green dots and samples from SUDs are shown as red dots. *p*-values were calculated using the Mann-Whitney test (**A**) for 93 samples analysis, while (**B**) had 57 samples analysis.

The microbial composition was also significantly different at the genus level among groups (Fig. 5A). *Thauera* (*p* < 0.0001), *Paracoccus* (*p* < 0.0001), and *Prevotella* (*p* = 0.0092) exhibited a relatively higher abundance in SUDs. *Anaerostipes* (*p* = 0.0015), *Bilophila* (*p* = 0.0020), *Clostridium XVIII* (*p* < 0.0001), *Dorea* (*p* = 0.0071), *Enterobacteriaceae_unclassified* (*p* = *0*.*0010*), *Fusicatenibacter* (*p* = 0.0026), *Gemmiger* (*p* = 0.0034), *Ruminococcus2* (*p* = 0.0057), and *Streptococcus* (*p* = 0.0053) were relatively more abundant in HC group. However, when comparing the 29 tested SUDs with the 28 age-matched HCs (Fig. 5B), *Anaerostipes* (*p* = 0.0284), *Bilophila* (*p* = 0.0388), *Clostridium XVIII* (*p* = 0.0011), *Enterobacteriaceae-unclassified* (*p* = 0.0179), *Fusicatenibacter* (*p* = 0.0145), *Gemmiger* (*p* = 0.0282), *Megasphaeram* (*p* = 0.0389), *Parabacteroides* (*p* = 0.004), *Paracoccus* (*p* < 0.0001), *Prevotella* (*p* = 0.0097), *Streptococcus* (*p* = 0.0046), and *Thauera* (*p* < 0.0001) at the genus level consistently differed between the 2 groups, while *Clostridium IV* (*p* = 0.0216) was only shown in the comparison between 29 SUDs and 28 age-matched HCs.

**Figure 6:**
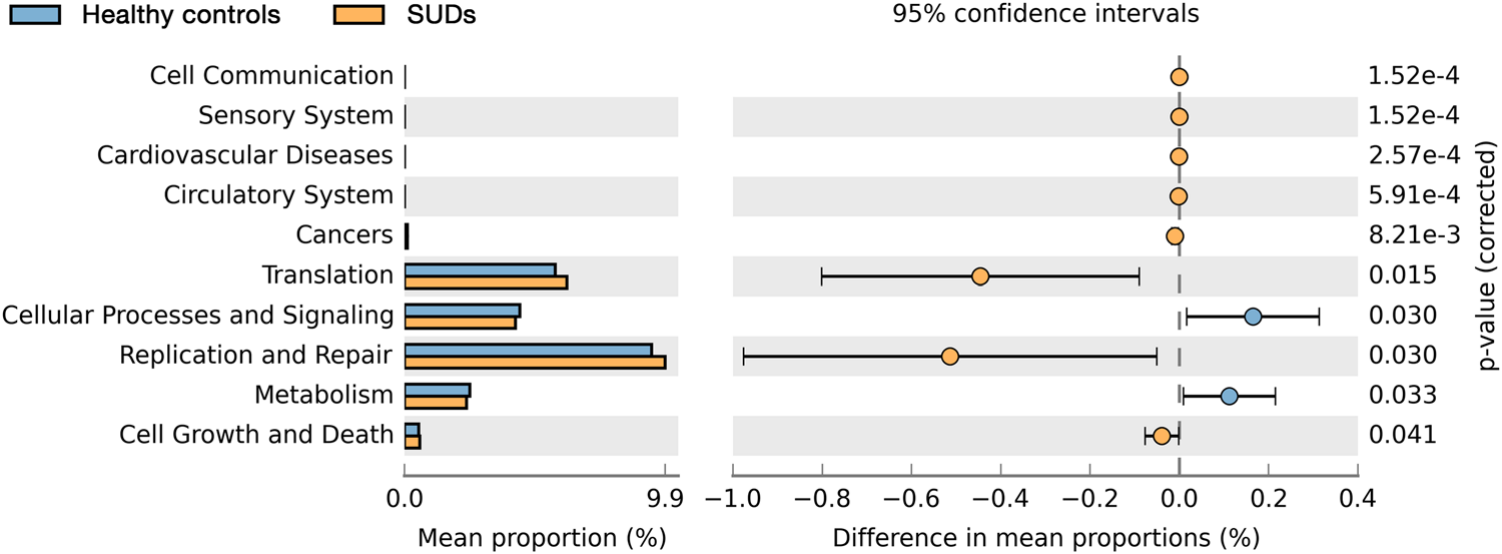
Compare the difference notability function in KEGG module prediction using 16S data with PICRUSt.

In addition, the functional diversity of the different putative metagenomes was assessed using the PICRUSt software^13^, which allows the prediction of metabolic pathways from the 16S rRNA reads. Pathways displaying a difference in mean proportions between SUDs and HCs at least 0.1% were represented (Fig. 6). Some pathways including translation (*p* = 0.015), DNA replication and repair (*p* = 0.03) and cell growth and death (*p* = 0.041) were over-represented in SUDs, whereas cellular processes and signaling (*p* = 0.03) and metabolism (*p* = 0.033) were under-represented in SUDs. These results indicate that factors associated with substance addiction may also influence the functional diversity, especially the metabolism of the different putative metagenomes.

## Discussion

A comprehensive and thorough investigation of the bacterial diversity of intestinal microbiota is essential for understanding their etiologies and for developing potential treatment strategies for SUDs in the future. The high-throughput sequencing has provided new insights into the compositions and structures of microbial communities. In this report, we used amplicon-sequencing to explore the bacterial diversity and the community structure in 93 fecal samples by sequencing the 16S rDNA hypervariable V3–V4 region. The V3–V4 region has been used in the study^14^ because it can provide a greater phylogenetic resolution and better analysis of the diversity and abundance.

We obtained 4,872,436 high-quality sequences with an average of 52,392 sequences per sample. At 97% identity, 148 OTUs belonging to 51 genera, 25 families, 16 orders, 12 classes, and 5 phyla were obtained, after filtering out the low-credibility OTUs and use Ribosomal Database Project (RDP) for assigning taxonomy to the sequences with a threshold 0.8~1. At the genus level, 51 different genera were identified in the samples, out of which 14 genera had abundances higher than 1% (Data not shown). We have shown that *Clostridium XI*, *Megasphaera*, *Barnesiella*, and *Blautia* were correlated with substance abuse and addiction regardless of the factor of age (Fig. 2). However, *Prevotellaceae-unclassified*, *Ruminococcaceae-unclassified*, *Porphyromonadaceae-unclassified*, and *Lachnospiraceae-unclassified* were only shown in the comparison of all 29 SUDs vs 28 HCs, but not identified in the comparison between age-matched groups. It is consistent with previous reports that some of these bacteria have been correlated with age^15^. In addition, certain bacteria gradually increase in abundance during substance use, thus they were regarded as potential biomarkers for the length of substance addiction. Furthermore, we screened potential biomarkers (*Thauera*, *Paracoccus*, and *Prevotella*) for substance addiction with LEfSe analysis. Therefore, our analyses proved adistinguished intestinal microbiota profile in SUDs, which provides a clue that may lead to strategies to identify or evaluate potential substance addicts.

To investigate whether there is an impact from bacterial function, PICRUSt was applied to the 16S rRNA deep sequencing data to infer bacterial metabolic functions. Interestingly, we found higher abundance of cell communication (*p* = 0.000152), cardiovascular disease (*p* = 0.000257), circulatory system (*p* = 0.000591), cell growth and death (*p* = 0.041), translation (*p* = 0.015) and replication and repair (*p* = 0.03) in SUDs comparing to HCs. Diseases in cardiovascular system, circulatory system and digestive system have been known as the consequences of substance addiction^16, 17^. Increased alpha diversity of the intestinal microbiota, as well as the enrichment of bacterial function related cardiovascular system, circulatory system and digestive system might reveal both harmful and protective bacterial effects on cardiovascular system, circulatory system and digestive system^18, 19^. Volpe *et al*. recently reported a characterization of the microbiome in cocaine users showed having a higher relative abundance of *Bacteroidetes* than in nonusers^20^. Kiraly *et al*. demonstrated in mice that perturbations of the gut microbiome affected behavioral response to cocaine in mice^2^. It is noticeable that intestinal microbiota in SUDs would change specifically with substance addiction, but not specific to any type of substance (Figure S1), suggesting a global switch of life style due to substance abuse in general could cause the significant change of intestinal microbiota in SUDs. It is also known that almost all patients with SUDs are involved in alcohol and tobacco addiction. Therefore, alcohol and tobacco use may also account for the large part of the effect. However, how substance abuse influences intestinal microbiota is still largely unknown. Further investigation is urgently needed to uncover the functions of intestinal microbiota in cardiovascular, circulatory and digestive systems in substance addicts.

In summary, amplicon-sequencing technology has greatly expanded our knowledge regarding the microbiota diversity and community structure in SUDs and HCs. We observed vast intestinal microbiota diversity with 4,872,436 high-quality sequences. Bacterial diversity in SUDs was higher than that in HCs and gradually increased with the length of substance abuse. More importantly, we identified *Thauera*, *Paracoccus*, and *Prevotella* as substance-related bacteria, although the specific functions of these bacteria require further testing and verification. Future analyses investigating the effects of anti-substance treatments on those substance addicts, using intestinal microbiota, would be valuable for developing new evaluation platforms to identify and treat these substance addicts.

## Methods

### Study Participants

Fecal samples were obtained from 50 SUDs and 51 HCs who come from Kunming Drug Rehabilitation Center. All subjects signed an informed consent form. The experimental protocol was approved by Medical Ethics Committee of the First Affiliated Hospital of Kunming Medical University and this study was conducted in accordance with the recommendations of the Medical Ethics Committee of the First Affiliated Hospital of Kunming Medical University. Inclusion criteria of subjects were as follows: an age of 19 ~ 46 years old (male), no incidence of antibiotics use within the three months prior to sampling. The detailed clinical parameters of the 101 participants are shown in the Table 1. Eight subjects were excluded from the study due to the intake of antibiotics before sampling. Exclusion criteria for all subjects were based on factors known or likely to impact the intestinal microbiota or known to be associated with microbial translocation^21^, including antibiotic or probiotic use with the previous 3 months, gastrointestinal morbidity, opportunistic infection, and evidence of human immunodeficiency virus, hepatitis B or C virus infection^22, 23^. HCs do not smoke or drink.

### Sample Collection, DNA extraction and 16S rRNA gene sequencing

Fecal sampling was performed 2 hours after breakfast. Samples were collected in a sterile container and immediately stored at −80 °C until further processing. Bacterial DNA was extracted using the QIAamp DNA Stool Mini Kit (Qiagen, USA) following manufacturer’s instructions. The adequacy of the amount of extracted DNA from the samples was verified with fluorometric quantitation (Qubit, Life Technologies, USA). Primers 5′CCTACGGGRSGCAGCAG3′ and 5′GGACTACVVGGGTATCTAATC3′ were used to amplify the V3–V4 hypervariable regions of 16S rDNA to comprehensively define the bacterial composition and abundance in a number of SUDs and HCs. Using Illumina HiSeq2500 platform, the 93 fecal samples yielded 4,872,436 high-quality reads (PE250) in total. On average, each sample yielded 52,392 sequences, ranging from 39,817 to 60,571. Clustering of all high-quality sequences at 97% identity resulted in 1691 OTUs, which were BLAST-searched against the RDP database for taxonomic assignments. After removing the low-credibility OTUs (together contributing only 8.9% of all sequences), a modified OTU table was obtained consisting of 148 OTUs with an average of 118 OTUs per sample (ranging from 62 to 166).

### Intestinal microbiota analysis

The Quantitative Insights Into Microbial Ecology pipeline was employed to process the sequencing data (QIIME ver. 1.9.0, http://qiime.org/). Briefly, raw sequences with exact matches to the barcodes were assigned to respective samples and identified as valid sequences whose primers and barcodes were trimmed for further quality control. Paired-end reads merged using PANDAseq^24^, sequences were de-noised using USEARCH (ver. 8.0.1623)^25^, and chimera checked with UCHIME^26^. Operational Taxonomic Units (OTUs) were picked using uclust at 97% similarity^25^, and representative sequences were generated. Sequences were aligned with PyNAST^27^ using Greengenes database and taxonomy assigned to the lowest possible taxonomic level using the Ribosomal Database Project Classifier at a 80% bootstrap value threshold^28^. OTUs found in above 50% samples were retained. The numbers of sequences were normalized for further analyses. Alpha-diversity indexes were compared in QIIME using a nonparametric two-sample t test, whereas *adonis* tests were used for beta-diversity comparisons. The upper limit of rarefaction depths (−e 39810) was used as the cut-off value. Metagenomic biomarker discovery use the online LEfSe program (http://huttenhower.sph.harvard.edu/galaxy/root/index)^29^. The function of intestinal microbiota was performed by online phylogenetic investigation of communities by reconstruction of unobserved states program (PICRUSt, http://picrust.github.io/picrust/). Functional modules were compared between SUDs and HCs with STAMP v2.1.3 (http://kiwi.cs.dal.ca/Software/STAMP) using Welch’s *t-test*. Statistical analyses were performed using *R* software package. GraphPad Prism was used to generate scatterplots of bacterial taxa.

### Statistics

The *p*-values are not corrected with FDR or Bonferroni in both LEfSe and PICRUSt.

## Electronic supplementary material

Supplemental information
