# Supplementary material for: Bacterial Diversity of Intestinal Microbiota in Patients with Substance Use Disorders Revealed by 16S rRNA Gene Deep Sequencing
Source: Sci Rep. 2017 Jun 15;7:3628. doi: 10.1038/s41598-017-03706-9 (PMC5472629; doi:10.1038/s41598-017-03706-9)
Supplement: Supplementary file 1 — Supplemental information [file 41598_2017_3706_MOESM1_ESM.pdf]

Bacterial Diversity of Intestinal Microbiota in Patients with Substance Use Disorders Revealed by 16S rRNA Gene Deep Sequencing

Yu Xu[1,2,3]*, Zhenrong Xie[1,3]*, Huawei Wang[1,3,4]*, Zongwen Shen[1,3], Youbing Guo[5], Yunhong Gao[5], Xin Chen[6], Qiang Wu[6], Xuejun Li[6], Kunhua Wang[1,2,3]

1 Yunnan Institute of Digestive Disease, the First Affiliated Hospital of Kunming Medical University, Kunming 650032,Yunnan, China;

2 Department of Gastrointestinal surgery, the First Affiliated Hospital of Kunming Medical University, Kunming 650032,Yunnan, China;

3 Kunming Engineering Technology Center of Diagnosis and Treatment of Digestive Diseases, Kunming 650032,Yunnan, China;

4 Department of reproduction and genetics, the First Affiliated Hospital of Kunming Medical University, Kunming 650032,Yunnan, China;

5 Yunnan Drug Enforcement Commission Office, Kunming 650032,Yunnan, China;

6 Yunnan Drug Enforcement Administration, Kunming 650032,Yunnan, China;

Correspondence: Kunhua Wang

Email: wangkunhua_group@163.com

*Three authors contributed equally to this work.

Table S1 Genus-level differences between 45 subjects substance use disorders (SUDs) and 48 healthy controls (HCs)

OTU	P-value	FDR	Bonferroni	SUDs mean (%)	HCs mean (%)
Prevotella	0.005	0.03	1	27.20	14.42
Bacteroides	0.03	0.12	1	24.20	33.97
Megamonas	0.10	0.27	1	9.64	8.86
Faecalibacterium	0.07	0.21	1	5.62	6.93
Phascolarctobacterium	0.17	0.36	1	5.37	4.15
Ruminococcus	0.45	0.59	1	3.97	1.64
Roseburia	0.74	0.84	1	2.94	2.16
Alloprevotella	0.0004	0.004	0.15	1.76	0.91
Alistipes	0.05	0.15	1	1.21	2.49
Clostridium XlVa	0.04	0.14	1	1.19	1.10
Parabacteroides	0.03	0.12	1	1.00	1.29
Escherichia/Shigella	0.52	0.65	1	0.99	1.65
Clostridium XI	0.68	0.78	1	0.77	1.83
Gemmiger	0.004	0.02	1	0.49	1.68
Blautia	0.10	0.27	1	0.44	0.48
Dialister	0.05	0.16	1	0.43	0.98
Haemophilus	0.18	0.37	1	0.37	0.80
Barnesiella	0.09	0.26	1	0.26	0.53
Megasphaera	0.04	0.13	1	0.23	0.70
Paraprevotella	0.95	0.99	1	0.19	0.72

Table S2 Genus-level differences between 29 age-matched SUDs and 28 age-matched HCs

OTU	P-value	FDR	Bonferroni	Age-matched SUDs mean (%)	Age-matched HCs mean (%)
Bacteroides	0.08	0.28	1	26.62	37.02
Prevotella	0.01	0.08	1	27.80	13.40
Megamonas	0.20	0.43	1	5.27	7.35
Faecalibacterium	0.40	0.52	1	6.60	6.41
Phascolarctobacterium	0.46	0.59	1	4.95	4.79
Escherichia/Shigella	0.53	0.66	1	1.10	2.45
Alistipes	0.29	0.45	1	1.17	2.22
Gemmiger	0.03	0.13	1	0.44	2.06
Roseburia	0.59	0.70	1	2.87	2.04
Parabacteroides	0.005	0.04	1	1.07	1.80
Ruminococcus	0.12	0.36	1	5.05	1.80
Clostridium XlVa	0.09	0.30	1	1.05	1.07
Dialister	0.13	0.36	1	0.47	1.00
Alloprevotella	0.02	0.10	1	1.73	0.99
Haemophilus	0.27	0.45	1	0.50	0.90
Paraprevotella	0.33	0.45	1	0.23	0.76
Veillonella	0.19	0.42	1	0.57	0.69
Blautia	0.13	0.36	1	0.36	0.55
Megasphaera	0.03	0.13	1	0.25	0.51
Parasutterella	0.04	0.16	1	0.19	0.47

Table S3 Genus-level differences among short-term SUDs (less than 12 months), Long-term SUDs (longer than 12 months), and HCs.

OTU	P-value	FDR	Bonferroni	Short-term SUDs mean (%)	Long-term SUDs mean (%)	HCs mean (%)
Bacteroides	0.07	0.22	1	31.18	21.36	33.97
Prevotella	0.01	0.07	1	18.26	30.83	14.42
Megamonas	0.25	0.51	1	13.14	8.22	8.86
Faecalibacterium	0.18	0.42	1	5.86	5.53	6.93
Phascolarctobacterium	0.38	0.57	1	5.36	5.37	4.15
Alistipes	0.08	0.24	1	0.54	1.49	2.49
Roseburia	0.84	0.88	1	3.67	2.64	2.16
Clostridium XI	0.88	0.91	1	1.38	0.53	1.83
Gemmiger	0.01	0.07	1	0.42	0.52	1.68
Escherichia/Shigella	0.74	0.83	1	0.73	1.10	1.65
Ruminococcus	0.56	0.74	1	2.45	4.59	1.64
Parabacteroides	0.04	0.14	1	0.41	1.23	1.29
Clostridium XlVa	0.09	0.26	1	1.76	0.96	1.10
Dialister	0.14	0.36	1	0.41	0.44	0.98
Alloprevotella	0.002	0.01	0.61	1.73	1.77	0.91
Haemophilus	0.40	0.59	1	0.99	0.12	0.80
Paraprevotella	0.85	0.88	1	0.14	0.21	0.72
Megasphaera	0.05	0.16	1	0.02	0.31	0.70
Barnesiella	0.24	0.49	1	0.14	0.31	0.53
Blautia	0.19	0.43	1	0.71	0.33	0.48

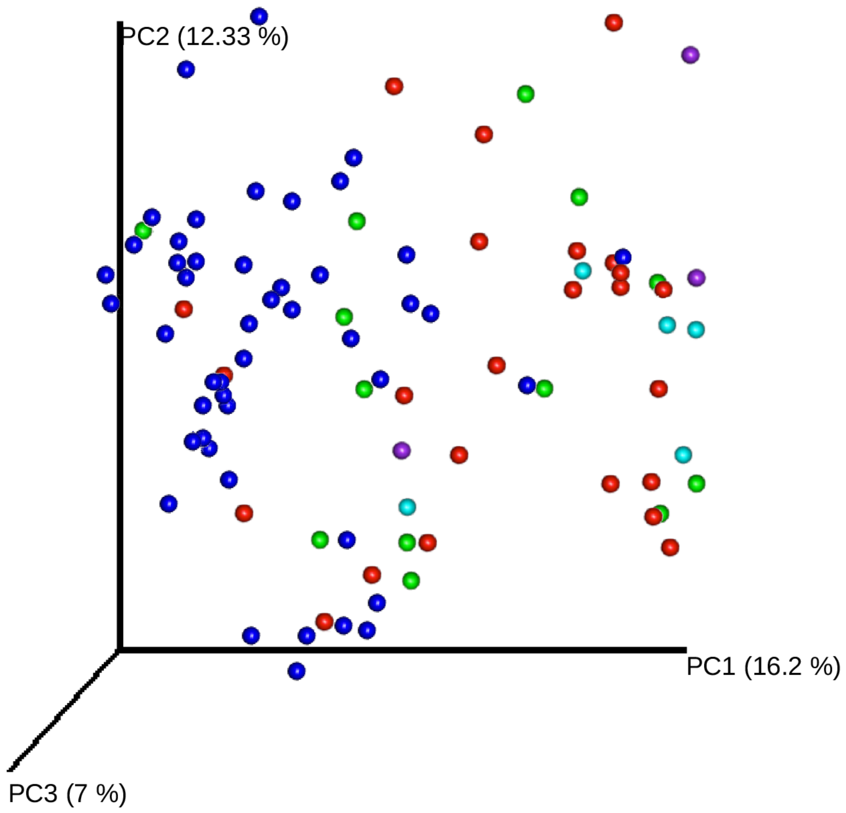

Figure S1 Beta diversity measures in different SUDs and HCs. PCoA is based on the data of unweighted-unifrac distances of samples, blue for the HCs, red for “heroin” group, green for “ice” group, purple for “ephedrine” group, cyan for “heroin+ice” group, and “heroin+ephedrine” group.

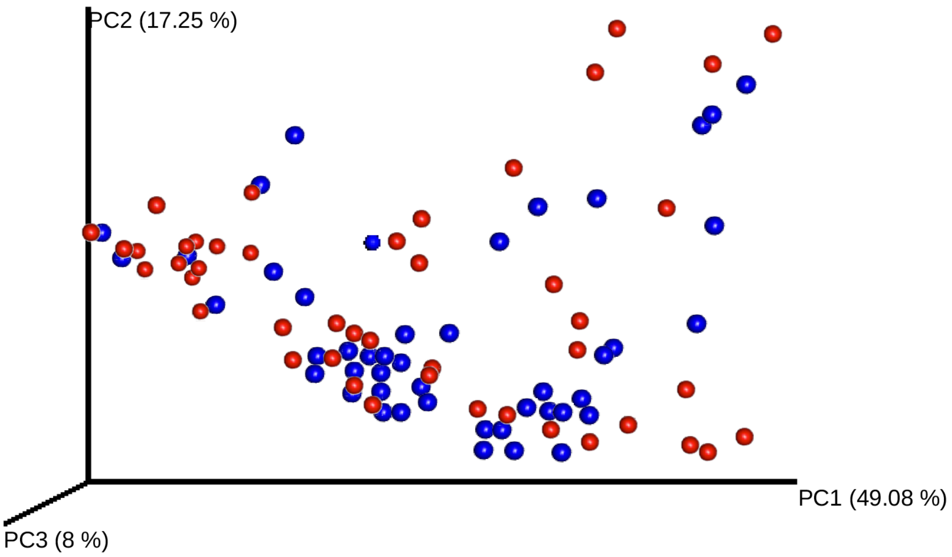

Figure S2 Beta diversity measures in SUDs versus healthy controls. Principal Coordinate Analysis (PCoA) is based on the data of weighted-unifrac distances of samples, blue for the healthy controls and red for the SUDs. ($R2$ =0.017, Pr (>F)=0.187).
